# Krϋppel‐like factor 15 suppresses renal glomerular mesangial cell proliferation via enhancing P53 SUMO1 conjugation

**DOI:** 10.1111/jcmm.16583

**Published:** 2021-05-04

**Authors:** Lingling Wu, Ou Li, Fengge Zhu, Xu Wang, Pu Chen, Guangyan Cai, Xiangmei Chen, Quan Hong

**Affiliations:** ^1^ Department of Nephrology First Medical Center of Chinese PLA General Hospital Nephrology Institute of the Chinese People's Liberation Army State Key Laboratory of Kidney Diseases National Clinical Research Center for Kidney Diseases Beijing Key Laboratory of Kidney Diseases Chinese PLA Institute of Nephrology Chinese PLA General Hospital Beijing China

**Keywords:** KLF15, Mesangial cell proliferation, P53, SUMO1

## Abstract

Mesangial cell (MC) proliferation is a key pathological feature in a number of common human renal diseases, including mesangial proliferative nephritis and diabetic nephropathies. Knowledge of MC responses to pathological stimuli is crucial to the understanding of these disease processes. We previously determined that Krϋppel‐like factor 15 (KLF15), a kidney‐enriched zinc‐finger transcription factor, was required for inhibition of MC proliferation. In the present study, we investigated the direct target gene and the underlying mechanism by which KLF15 regulated mesangial proliferation. First, we screened small ubiquitin‐related modifier 1 (SUMO1) as the direct transcriptional target of KLF15 and validated this finding with ChIP‐PCR and luciferase assays. Furthermore, we demonstrated that overexpressing KLF15 or SUMO1 enhanced the stability of P53, which blocked the cell cycle of human renal MCs (HRMCs) and therefore abolished cell proliferation. Conversely, knockdown of SUMO1 in HRMCs, even those overexpressed with KLF15, could not inhibit HRMC proliferation rates and increase SUMOylation of P53. Finally, the results showed that the levels of SUMOylated P53 in the kidney cortices of anti‐Thy 1 model rats were decreased during proliferation periods. These findings reveal the critical mechanism by which KLF15 targets SUMO1 to mediate the proliferation of MCs.

## 1 INTRODUCTION

Mesangial cells (MCs) account for approximately one‐third of the cell population within the glomerulus. They play important roles in homeostasis by maintaining the structural architecture of the glomerulus, producing and maintaining the mesangial matrix, regulating the filtration surface area and phagocytosing apoptotic cells or immune complexes.^1^ In many glomerular diseases, such as mesangial proliferative nephritis and diabetic nephropathies, MCs are injured and undergo a major change in phenotype, resulting in uncontrolled mesangial proliferation and excessive deposition of mesangial matrix.^2^, ^3^, ^4^ In addition to hyperproliferating and increasing their production of extracellular matrix (ECM) proteins and matrix metalloproteinases, MCs can release inflammatory factors, resulting in glomerular sclerosis and interstitial fibrosis; further aggravate renal damage; and cause progression to irreversible end‐stage renal disease.^5^, ^6^, ^7^ Knowledge of the MC response to pathological stimuli is crucial for the development of treatments to reduce the kidney damage caused by abnormal proliferation of MCs and delay progression into end‐stage renal disease.

The Krüppel‐like factors (KLFs) are a group of zinc‐finger DNA‐binding transcription factors. Increasing evidence indicates that this family is involved in a variety of cellular processes, such as cell differentiation, cardiac remodelling, haematopoiesis and stem cell fate determination.^8^, ^9^, ^10^ The KLF family consists of eighteen members, among which KLF15 is widely distributed in the kidney, pancreas, heart, liver and skeletal muscles. Previous studies have demonstrated that KLF15 is a key transcriptional regulator in diverse physiological processes, including gluconeogenesis, immune responses and adipocyte differentiation.^11^, ^12^, ^13^, ^14^ Our previous study has demonstrated that KLF15 is required for inhibiting the proliferation of MCs.^15^ This study aimed to clarify the direct target gene and the downstream mechanism by which KLF15 regulates mesangial proliferation.

## 2 MATERIALS AND METHODS

### 2.1 Anti‐Thy1 nephritis model

Male Wistar rats (Beijing Vital River Laboratory Animal Technology Co., Ltd.) weighing between 200 and 220 g were randomly allocated to the control and anti‐Thy1 groups. All rats were housed in an animal care facility under a light/dark cycle of 12/12 hours with free access to food and water. Welfare‐related assessments and interventions were performed throughout the experiment. In total, 28 rats were injected with a mouse anti‐Thy1 monoclonal antibody, as described previously,^16^ and seven were injected with saline (controls). Glomeruli were purified from the renal cortex tissue using a sieving method. Sera were collected for measurements of serum urea nitrogen and creatinine levels. Anti‐Thy1 model rats were killed on days 0, 3, 5, 7 and 10, and their glomeruli were harvested. All animal welfare and experimental procedures were performed in strict accordance with the Guide for the Care and Use of Laboratory Animals (USA National Research Council, 1996).

### 2.2 Human renal MC (HRMC) culture and treatment

Primary renal HRMCs were purchased from ScienCell (San Diego, CA). HRMCs from the third to sixth passages were maintained in MC Medium (MsCM; ScienCell) according to the manufacturer's instructions. For experiments, the cells were grown to confluence, growth‐arrested in reduced serum (0.5% FBS) for 24 hours and split into different experimental groups.

For treatment with different concentrations of glucose, the cells were incubated in MsCM containing either 5 mmol/L glucose (normal glucose) or 30 mmol/L glucose (high glucose, HG) for 48 hours at 37°C. Cells incubated under identical experimental conditions with 5 mmol/L glucose and 25 mmol/L mannitol instead of 30 mmol/L glucose were used as osmotic controls. For treatment with PDGF‐BB, the cells were incubated in MsCM containing 20 ng/mL PDGF‐BB (R&D Systems, Minneapolis, MN) for 48 hours at 37°C.

### 2.3 Immunohistochemistry (IHC)

Paraffin‐embedded kidney tissue was sectioned at 4 μm thickness, and the sections were stained for IHC. The slices were incubated with 3% H_2_O_2_ to block endogenous peroxidase following deparaffinization. After antigen retrieval and blocking, the slices were incubated with anti‐KLF15 (ab81604, Abcam), anti‐small ubiquitin‐related modifier 1 (SUMO1, ab32058, Abcam) and anti‐PCNA (ab92552, Abcam) antibodies at 4°C. The slices were then incubated with a secondary antibody labelled with horseradish peroxidase at 37°C for 30 minutes, and the immunohistochemical reaction was observed according to the manufacturer's instructions with 3,3’‐diaminobenzidine (DAB, ORIGENE, CN) as the chromogenic agent. Subsequently, the slices were counterstained with haematoxylin‐eosin and imaged with a light microscope (Olympus). Immunohistochemical staining was evaluated with Image J software according to the average optical density (AOD) value.

### 2.4 Chromatin immunoprecipitation (ChIP) with parallel sequencing (ChIP‐Seq)

ChIP was performed using a Simple ChIP Plus Enzymatic Chromatin IP Kit (Magnetic Beads; Cell Signaling Technology) according to the manufacturer's protocol. Briefly, HRMCs were overexpressed with either KLF15 or scramble at a cell density of 2.0 × 10^5^ cells/mL and cross‐linked with 1.7% formaldehyde in PBS for 5 minutes at room temperature (RT). Cross‐linked cells were lysed to obtain the nuclear fraction, which was sonicated in 1 × ChIP buffer. The lysates were clarified by centrifugation at 9300 × *g* for 10 minutes at 4°C. Chromatin samples were incubated with either an anti‐KLF15 antibody (ab81604, Abcam) or an anti‐IgG antibody (background control) overnight at 4°C. The antibody‐bound complexes were captured by incubation with protein G magnetic beads. ChIP‐Seq libraries for sequencing were prepared using a TruSeq ChIP Sample Prep Kit (Illumina). The libraries were subjected to parallel sequencing with a HiSeq2500 sequencer (Illumina) using the single‐end 50 bp sequencing length protocol. Next‐generation sequencing (NGS) raw data were converted into FASTQ files using CASAVA software (version 1.8.2), and each data set was aligned to the human reference genome (UCSC hg19) using the Burrows‐Wheeler Aligner (version 0.7.12).^17^ ChIP‐Seq peak calling was performed using the MACS2 program (version 2.0.1) with the default parameters but a ‐q value of 0.05, with the input data for subtraction.^18^ Peak comparison with the scramble control (the parental cells) and overexpression samples was performed with Diffbind, an R package, using the DeSeq2 algorithm, and a false discovery rate of 0.1 was considered to indicate significance.^19^ Superenhancers were identified from the set of peaks detected in DMSO‐treated HRMCs with the superenhancer software ROSE.^20^ The ChIP‐Seq peaks were annotated with the R package ChIPpeakAnno, and promoters were defined as KLF15‐enriched regions within 1 kb of the transcription start site.^21^ The gene with the transcription start site closest to the centre of each superenhancer was defined as the target gene of that superenhancer.

### 2.5 ChIP‐quantitative real‐time PCR (ChIP‐PCR)

The ChIP‐PCR procedure was similar to the procedure described above. ChIP DNA from anti‐KLF15 antibody‐treated cells was used to detect the association between KLF15 and SUMO1. DNA from anti‐IgG antibody‐treated cells served as the control. Purified DNA was used for analysis of the SUMO1 proximal promoter region by real‐time PCR on an ABI PRISM 7600 using SYBR GreenER qPCR Supermix. The SUMO1 primers were as follows: forward: 5′‐AGCAGCGGTCTTTAGCATCA‐3′; reverse: 5′‐TCGGTTAACCAGCACCCTTG‐3′. The relative amplification of the promoter sequence of the gene was calculated using the 2^−ΔΔCT^ method, and normalization was performed against a 1:100 dilution of the input DNA.

### 2.6 Cell labelling and stable isotope labelling by amino acids in cell culture (SILAC) analysis

Cells at 15% confluency were seeded in the appropriate complete medium (for HRMCs: MsCM). The labelling and SILAC procedures have been described in previous research.^22^ Briefly, after being labelled with light (^13^C_6_‐labelled) or heavy (^15^N_2_‐labelled) lysine and light (^13^C_6_‐labelled) or heavy (^15^N_4_‐labelled) arginine, cells were transfected with a KLF15 overexpression plasmid or a control plasmid. After treatment, the cells were trypsinized and counted to obtain a cell pellet of 2 × 10^7^ cells/condition, and the cells were subjected to SILAC analysis using mass spectrometry. The heavy and light cell pellets were lysed in radioimmunoprecipitation buffer spiked with protease and phosphatase inhibitors using short 15 second sonication bursts. The lysates were centrifuged at 14,000 rpm for 20 minutes. After centrifugation, the supernatants were collected, and the protein concentration was measured using a Hitachi L‐8900 amino acid analyser. Aliquots of 200 μg of protein were prepared from the samples, combined and precipitated using a methanol‐chloroform precipitation method. The protein pellets were resuspended in 8 mol/L urea/0.4 mol/L ammonium bicarbonate buffer, reduced with 45 mmol/L DTT for 30 minutes at 37°C, alkylated with 100 mmol/L iodoacetamide for 30 minutes in the dark at RT and digested with Lys‐C protease (1:20 w/w) overnight (~16 hours) at 37°C. The Lys‐C digest was further diluted and digested with trypsin (1:20 w/w) for 8 hours at 37°C. The digest was desalted with a MacroSpin column (The Nest Group, Inc) and dried in a SpeedVac concentrator. The desalted peptides were then enriched with phosphopeptides using titanium dioxide resin embedded in 10 μL tips (Glygen Corp). The flowthroughs were reserved, and the enriched peptides were eluted using a 1:33 ratio of ammonium hydroxide to water. The SpeedVac‐dried flowthrough and elution fractions were resuspended in buffer A (0.1% formic acid in water) and subjected to liquid chromatography‐tandem mass spectrometry (LC/MS) analysis.

### 2.7 Dual‐luciferase reporter assay

Wild‐type human SUMO1(NM_001005781) promoter with 1604bp (−1474nt ~ +129nt) including the potential binding site (−205nt ~ −199nt) was obtained using PCR amplify and subcloned into pGL3‐Basic vector (Cat.# E1751 Promega) with Sac I and Bgl II restriction enzyme sites, named pGL3‐WT‐SU. Also, the binding site ‐CACCCA‐ within the promoter of SUMO1 was mutated to ‐CGTTTG‐, named pGL3‐MT‐SU. Plasmid (pReceiver‐M94) contained the human KLF15 full‐length cDNA was obtained from GeneCopoeia. The KLF15 overexpression plasmid and pGL3‐WT‐SU or pGL3‐MT‐SU were transfected into HEK293T cells with Lipofectamine 2000 reagent. The samples were then cotransfected with a pRL‐TK (Cat #E2241, Promega) plasmid expressing Renilla luciferase. After 24 hours of transfection, the cells were lysed. Firefly and Renilla luciferase activity levels were examined using a dual‐luciferase reporter assay system (Biotek).

### 2.8 RNA preparation, cDNA synthesis and RT‐qPCR analysis

Total RNA was extracted using TRIzol (Invitrogen) and purified using a RNeasy Mini Kit (Qiagen) according to the manufacturer's instructions. cDNA was generated using a ProtoScript first‐strand cDNA synthesis kit (New England Biolabs). Quantitative PCR was performed using Power SYBR Green Master Mix (Life Technologies). The oligonucleotide sequences used for RT‐qPCR are provided in Table 1.

**TABLE 1:** Primers for RT‐qPCR analysis

Primer	Sequence (5'→3')
KLF15	Forward: GGGCCAAGGCCAGAACTTTA
	Reverse: GGCCTACTTCCTTCTCCTCC
SUMO1	Forward: TGTCAAAGACAGGGTGTTCCA
	Reverse: CCTCCATTCCCAGTTCTTTTGG
18s RNA	Forward: GTAACCCGTTGAACCCCATT
	Reverse: CCATCCAATCGGTAGTAGCG

### 2.9 Cell proliferation assay

Human full‐length SUMO1 cDNA (NM_003352.8) obtained from OriGene Technologies (Beijing, China) was inserted into pCMV6‐Entry plasmid (CAT#: RC200633). Cell proliferation was analysed with a Click‐iT® Plus EdU Alexa Fluor® 555 Imaging Kit (Invitrogen) according to the manufacturer's instructions. In brief, cells were incubated in a 6‐well culture plate and transfected with a KLF15 control plasmid (Scramble, the VECTOR group), a KLF15 overexpression plasmid (the same above, the KLF15 group), an expression vector for SUMO1 (the VECTOR group), a SUMO1 overexpression plasmid (the SUMO1 group), negative control siRNA (the SI CON group), SUMO1 siRNA (the SI SUMO1 group), a KLF15 overexpression plasmid with SUMO1 siRNA (the KLF15 + SI SUMO1 group) or a KLF15 overexpression plasmid with negative control siRNA (the KLF15 + SI CON group) for 24 hours. Then, the medium was replaced with medium supplemented with 20 ng/mL PDGF‐BB for 48 hours, and an optimized EdU concentration of 10 μmol/L was added about 12 hours before PDGF‐BB stimulation. After treatment, the cells were fixed with 4% formaldehyde, permeabilized with 0.5% Triton X‐100, washed with 3% BSA in PBS, incubated with Click‐iT® reaction cocktail for 30 minutes at RT and incubated with DAPI reaction buffer for 10 minutes at RT while protected from light. The cells were observed by fluorescence microscopy (Olympus, Tokyo, Japan). The nuclei of proliferating cells were then stained red.

### 2.10 Western blot analysis and immunoprecipitation (IP)

Cells were lysed using RIPA buffer (Thermo Scientific), and the total protein in the supernatants was quantified using a BCA protein assay kit (Thermo Scientific). Western blot analysis and IP were performed as described previously.^23^, ^24^ Anti‐KLF15 antibody (AV32587, Merk), anti‐SUMO1 antibody(S8070, Merk) and anti‐β‐actin (A5316, Sigma) were used for Western blot analysis. Anti‐P53 antibody (P5813, Merk) was used for IP analysis.

### 2.11 Bioinformatics analysis

Candidates from ChIP‐Seq or SILAC showed at least twofold enrichment over the controls. Gene Ontology (GO) analysis was performed with the Database for Annotation, Visualization and Integrated Discovery (DAVID; version 6.7). P‐values were adjusted for multiple hypothesis testing using the Benjamini‐Hochberg method. Significantly enriched categories in the function subontology and KEGG pathways with an adjusted *P*‐value <.05 were chosen. Ingenuity Pathway Analysis (IPA) of 52 intersecting genes or proteins between ChIP‐Seq and SILAC was performed on the Qiagen IPA Platform (https://digitalinsights.qiagen.com/), and the motifs in the target genes were analysed using MEME Suite 5.3.0. (http://meme‐suite.org/).

### 2.12 Statistical analysis

All experiments were performed in triplicate, and the results are expressed as the means ± *SE*s. All data were analysed using SPSS software (ver. 20.0; SPSS Inc) and compared using Student's *t* test or one‐way ANOVA. A *P*‐value <.05 was considered to reflect statistical significance.

## 3 RESULTS

### 3.1 Screening of KLF15‐binding genes through ChIP‐Seq in primary renal glomerular MCs

**FIGURE 1:**
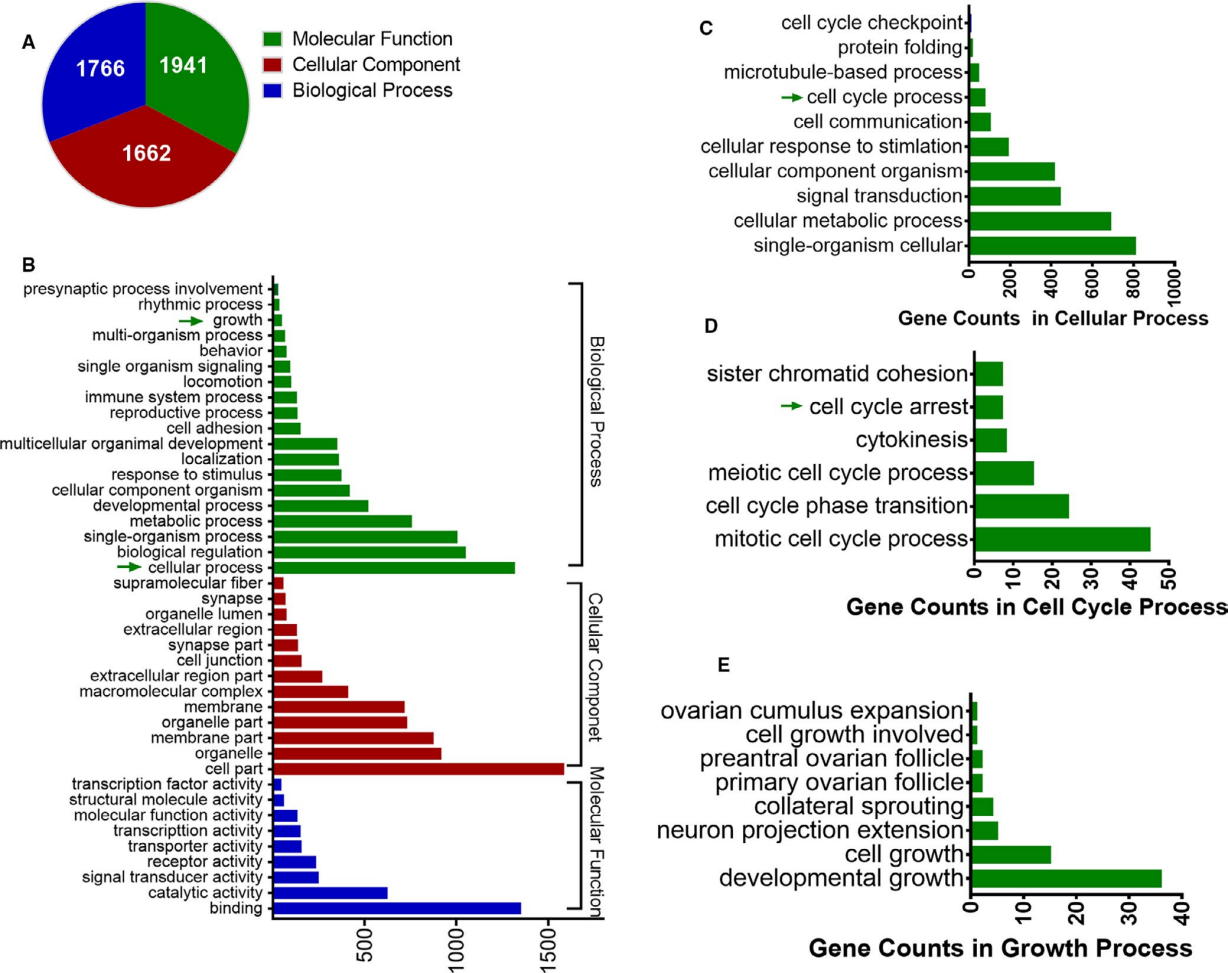
*Annotation of KLF15 ChIP‐Seq data in HRMCs*. A‐B, GO analysis of the 2,478 screened genes. C, GO analysis of 1,315 cell process‐related genes. D, Cell cycle process‐related gene analysis results. E, Growth process‐related gene analysis results

To identify the direct binding partner genes of KLF15, we performed ChIP‐Seq analysis and ultimately screened 2478 genes. GO analysis of these genes through the tool at the website www.uniprot.org (Figure 1A,B) revealed molecular function terms associated with 1941 genes, cellular component terms related to 1662 genes and biological process terms related to 1766 genes. Since our aim was to find out how KLF15 affects MCs, we focused on cell process‐related genes. Among the 1315 cell process‐related genes, 74 genes were found to participate in cell cycle processes; specifically, these genes participated in the mitotic cell cycle, the cell cycle phase transition and other processes (Figure 1C,D). Furthermore, we analysed the genes involved in growth and found that they were related to developmental growth, cell growth and other types of growth (Figure 1E).

### 3.2 Screening of differentially regulated genes in KLF15‐overexpressing renal glomerular MCs using SILAC and LC/MS

**FIGURE 2:**
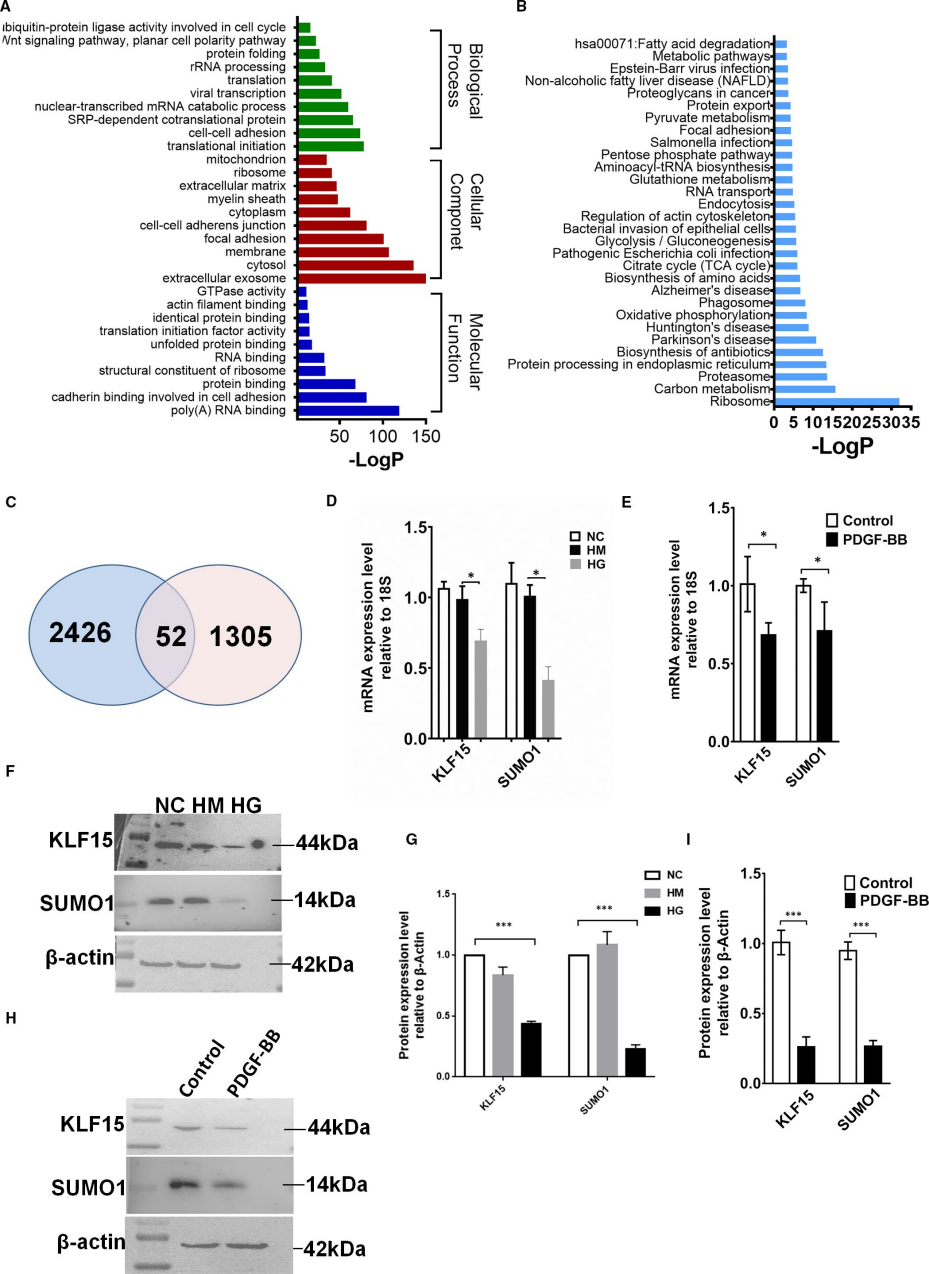
*Bioinformatics analysis of KLF15‐binding genes*. A, DAVID analysis and IPA were performed to acquire the GO domains of the 1,357 SILAC‐LC/MS‐screened proteins. B, Top 30 signalling pathways identified by IPA of the SILAC‐LC/MS data. C, Fifty‐two genes identified by bioinformatics analysis of ChIP‐Seq data and SILAC‐LC/MS data. D, KLF15 and SUMO1 mRNA expression in HG‐treated HRMCs. E, KLF15 and SUMO1 mRNA expression in PDGF‐BB‐treated HRMCs. F‐G, KLF15 and SUMO1 protein expression in HG‐treated HRMCs. H‐I, KLF15 and SUMO1 protein expression in PDGF‐BB‐treated HRMCs. *P < .05, ***P < .01, n = 3

SILAC and LC/MS analysis of HRMCs overexpressing KLF15 compared to parental cells led to the identification of 1357 proteins. We used the DAVID and IPA to acquire the GO domains and enriched pathways of the quantified proteins identified by SILAC. Interestingly, many proteins biological function‐related terms were among the top 30 significantly enriched GO terms, including the regulation of cellular amino acid metabolic process, proteasome complex, protein binding, and transcription and translation terms, all of which are closely related to ubiquitination (Figure 2A). Figure 2B shows the top 30 significantly enriched pathway terms. Several biological process terms, including the proteasome and neurodegenerative disease terms, were also related to ubiquitination.

### 3.3 Bioinformatics analysis of KLF15‐binding genes that are potentially associated with renal glomerular MC proliferation

To explore the KLF15‐binding genes that are potentially associated with renal glomerular MC proliferation, we performed bioinformatics analysis of the ChIP‐Seq data and the SILAC‐LC/MS data. Fifty‐two genes were screened (Table 2 and Figure 2C), and five of these genes, including SUMO1, macrophage migration inhibitory factor (MIF), eukaryotic initiation factor (EIF), insulin‐like growth factor (IGF) and reticulocalbin (RCN), were closely related to cell proliferation. Since the GO and pathway analyses of the differentially expressed proteins suggested that the screened genes were related mainly to the ubiquitination process, we concluded that SUMO1, a member of the ubiquitin‐like (UBL) protein family, participates in post‐translational modification similar to ubiquitination as the target gene of KLF15.

**TABLE 2:** 52 genes of bioinformatics analysis of ChIP‐Seq data and SILAC‐LC/MS data

Refseq	Gene Symbol	Description	Fold Change	Up/Down
NM_002940	ABCE1	ATP‐binding cassette, subfamily E (OABP), member 1	0.666	Down
NM_002197	ACO1	aconitase 1, soluble	0.584	Down
NM_005100	AKAP12	A kinase (PRKA) anchor protein 12	1.536	Up
NM_000477	ALB	albumin	0.297	Down
NM_001627	ALCAM	activated leukocyte cell adhesion molecule	1.502	Up
NM_002860	ALDH18A1	aldehyde dehydrogenase 18 family, member A1	1.532	Up
NM_000700	ANXA1	annexin A1	1.941	Up
NM_004039	ANXA2	annexin A2	1.506	Up
NM_000701	ATP1A1	ATPase, Na+/K+ transporting, alpha 1 polypeptide	1.520	Up
NM_004281	BAG3	BCL2‐associated athanogene 3	0.404	Down
NM_001221	CAMK2D	calcium/calmodulin‐dependent protein kinase II delta	1.695	Up
NM_001344	DAD1	defender against cell death 1	1.763	Up
NM_001539	DNAJA1	DnaJ (Hsp40) homolog, subfamily A, member 1	1.888	Up
NM_012100	DNPEP	aspartyl aminopeptidase	1.648	Up
NM_001415	EIF2S3	eukaryotic translation initiation factor 2, subunit 3 gamma, 52 kDa	1.731	Up
NM_001568	EIF3E	eukaryotic translation initiation factor 3, subunit E	1.726	Up
NM_021814	ELOVL5	ELOVL fatty acid elongase 5	1.650	Up
NM_014673	EMC2	ER membrane protein complex subunit 2	1.917	Up
NM_006832	FERMT2	fermitin family member 2	0.487	Down
NM_000583	GC	group‐specific component (vitamin D binding protein)	0.323	Down
NM_000518	HBB	haemoglobin, beta	0.592	Down
NM_000521	HEXB	hexosaminidase B (beta polypeptide)	1.603	Up
NM_002138	HNRNPD	heterogeneous nuclear ribonucleoprotein D	0.440	Down
NM_001553	IGFBP7	insulin‐like growth factor‐binding protein 7	1.708	Up
NM_005578	LPP	LIM domain containing preferred translocation partner in lipoma	1.998	Up
NM_000240	MAOA	monoamine oxidase A	0.589	Down
NM_005909	MAP1B	microtubule‐associated protein 1B	0.521	Down
NM_013283	MAT2B	methionine adenosyltransferase II, beta	1.714	Up
NM_002395	ME1	malic enzyme 1, NADP(+)‐dependent, cytosolic	0.449	Down
NM_002415	MIF	macrophage migration inhibitory factor (glycosylation‐inhibiting factor)	1.775	Up
NM_005956	MTHFD1	methylenetetrahydrofolate dehydrogenase (NADP + dependent) 1	1.559	Up
NM_002526	NT5E	5'‐nucleotidase, ecto (CD73)	1.641	Up
NM_013341	OLA1	Obg‐like ATPase 1	0.596	Down
NM_002583	PAWR	PRKC, apoptosis, WT1, regulator	1.776	Up
NM_013374	PDCD6IP	programmed cell death 6 interacting protein	0.400	Down
NM_004911	PDIA4	protein disulphide isomerase family A, member 4	1.692	Up
NM_003681	PDXK	pyridoxal (pyridoxine, vitamin B6) kinase	1.897	Up
NM_021105	PLSCR1	phospholipid scramblase 1	1.684	Up
NM_002734	PRKAR1A	protein kinase, cAMP‐dependent, regulatory, type I, alpha	0.464	Down
NM_012293	PXDN	peroxidasin homolog (Drosophila)	1.658	Up
NM_012414	RAB3GAP2	RAB3 GTPase activating protein subunit 2 (non‐catalytic)	1.765	Up
NM_002901	RCN1	reticulocalbin 1, EF‐hand calcium‐binding domain	0.562	Down
NM_001032	RPS29	ribosomal protein S29	1.510	Up
NM_016106	SCFD1	sec1 family domain containing 1	1.683	Up
NM_014766	SCRN1	secernin 1	0.303	Down
NM_021199	SQRDL	sulphide quinone reductase‐like (yeast)	1.903	Up
NM_003352	SUMO1	small ubiquitin‐like modifier 1	2.154	Up
NM_004607	TBCA	tubulin folding cofactor A	0.554	Down
NM_006759	UGP2	UDP‐glucose pyrophosphorylase 2	0.450	Down
NM_016226	VPS29	vacuolar protein sorting 29 homolog (S. cerevisiae)	1.811	Up
NM_006826	YWHAQ	tyrosine 3‐monooxygenase/tryptophan 5‐monooxygenase activation protein, theta	0.554	Down
NM_080660	ZC3HAV1L	Zinc‐finger CCCH‐type, antiviral 1‐like	0.552	Down

Increasing amounts of evidence have indicated that HG is one of the major factors inducing the development of diabetic nephropathy, and it promotes MC proliferation and increased matrix synthesis in vitro.^25^ A previous study has shown that PDGF‐BB is essential for MC proliferation preceding the development of glomerulosclerosis in experimental glomerulonephritis.^26^ After confirming that MCs were stimulated by HG or PDGF‐BB in vitro, we detected changes in the expression of KLF15 and SUMO1 at both the RNA and protein levels and found that these molecules had similar trends (Figure 2D‐I). Finally, we determined SUMO1 to be the target gene of KLF15.

### 3.4 KLF15 up‐regulates SUMO‐1 gene expression by binding to a 16 bp CACCCA‐SUMO‐1 promoter region and a C+A‐rich motif

**FIGURE 3:**
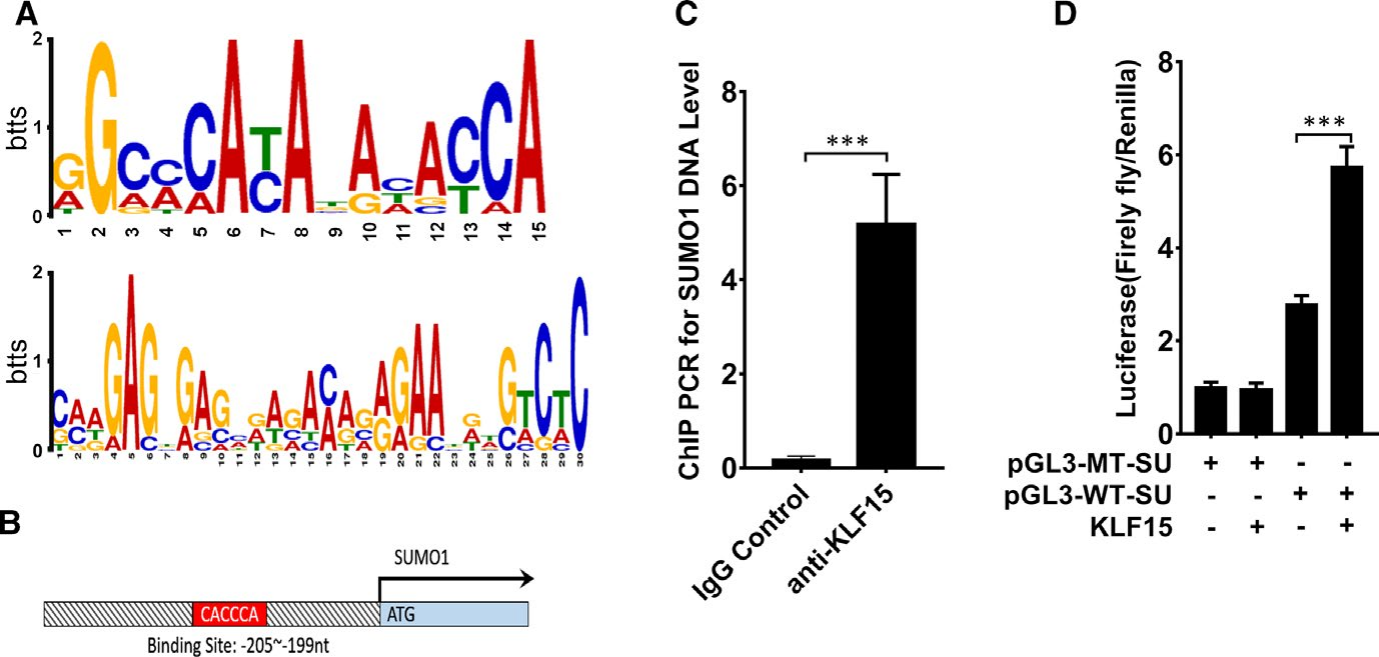
*Motif analysis and validation of KLF15‐binding sites*. A, Consensus KLF15‐binding motif identified by WebLogo in the KLF15 ChIP‐Seq data using the MEME program. B, Target region of the SUMO1 promoter to which KLF15 binds. C, ChIP‐PCR results for SUMO1, n = 5. D, Dual‐luciferase reporter assay results, n = 5. ***P < .01

We used the MEME suite (http://meme‐suite.org/tools/meme) to characterize the KLF15‐binding motifs of the 52 screened genes from the KLF15 ChIP‐Seq data and identified the KLF‐binding motif (Figure 3A). In addition, we further analysed more than one thousand bases upstream of the SUMO1 promoter and found that KLF15 could recognize and bind to a 16 bp sequence (nucleotides −205 ~ −199) including ‐CACCCA‐ (Figure 3B). ChIP‐PCR confirmed that KLF15 bound to the SUMO1 promoter, and the results showed significantly higher expression of SUMO1 in the anti‐KLF15 antibody group than in the background control group (Figure 3C). Furthermore, we performed a dual‐luciferase reporter assay to confirm the targeting relationship between SUMO1 and KLF15. The wild‐type SUMO1 promoter group showed higher luciferase activity than the pGL3 vector and mutant groups in HRMCs, and the activity was significantly increased by overexpression of KLF15. The enhancement in luciferase activity was reversed by transfection with a plasmid expressing the mutant promoter region (Figure 3D). Taken together, these data suggest that SUMO1 is a direct transcriptional target of KLF15.

### 3.5 Screening of P53 as the SUMOylation substrate of SUMO1

**FIGURE 4:**
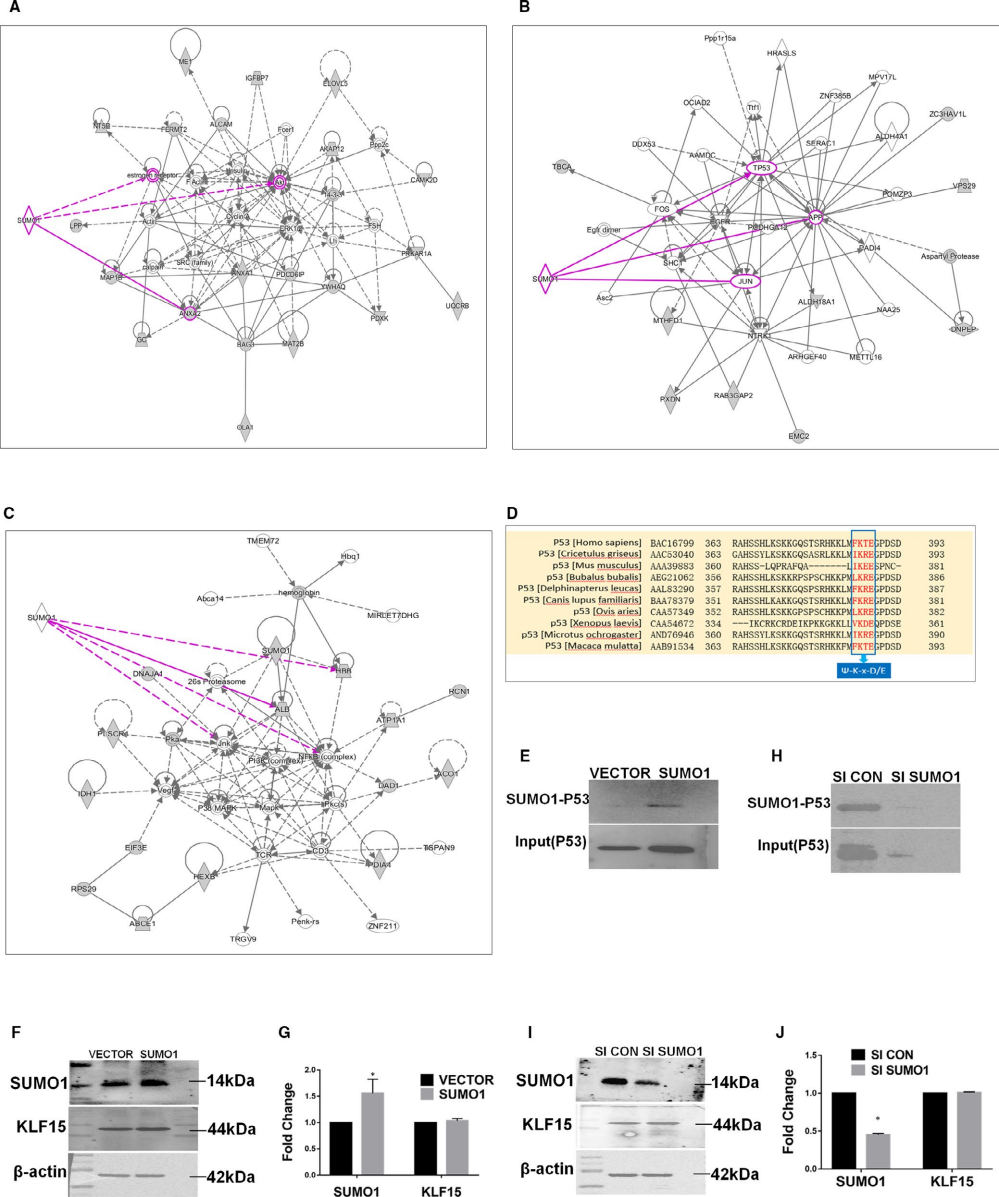
*IPA of downstream signalling molecules of SUMO1 and validation*. A‐C, IPA network associated with SUMO1. D, Alignment of various P53 sequences from different species (indicated). The SUMOylation consensus sites are shown in red. E‐J, SUMO1‐P53, KLF15 and SUMO1 expression in SUMO1‐overexpressing or SUMO1‐depleted HRMCs. *P < .05, **P < .01, n = 3

SUMO modifications are widely expressed post‐translational modifications in eukaryotes. Reversible conjugation of these modifications to substrate proteins is also known as SUMOylation, which plays important roles in regulating the functions of the target proteins and further participates in various biological processes, such as nucleocytoplasmic transport, transcriptional regulation, apoptosis, protein stabilization, stress responses and cell cycle progression.^27^ To explore the downstream signalling molecules directly conjugated by SUMO1, we performed network analysis of SUMO1 using the IPA database, and the data showed that SUMO1 could directly conjugate to P53, APP, JUN and AKT, among other proteins (Figure 4A‐C). We initially selected P53 as a downstream molecule of SUMO1 because it is a well‐known protein that is closely related to cell proliferation.^28^, ^29^ Furthermore, we used SUMOsp software to predict the possible SUMOylation sequences of P53 in various species and found that P53 had the SUMOylation sequence Ψ‐K‐x‐D/E (Figure 4D). To determine whether P53 is indeed modified by SUMOylation, we transiently transfected HRMCs with a SUMO1 overexpression plasmid or SUMO1 siRNA. Both the IP and Western blot results revealed trends in expression changes of SUMO1‐P53 and P53 that were consistent with the changes in SUMO1 (Figure 4E‐J). These data indicate that P53 is a SUMOylation substrate of SUMO1.

### 3.6 KLF15 inhibits MC proliferation by promoting SUMO1 expression and P53 SUMOylation

**FIGURE 5:**
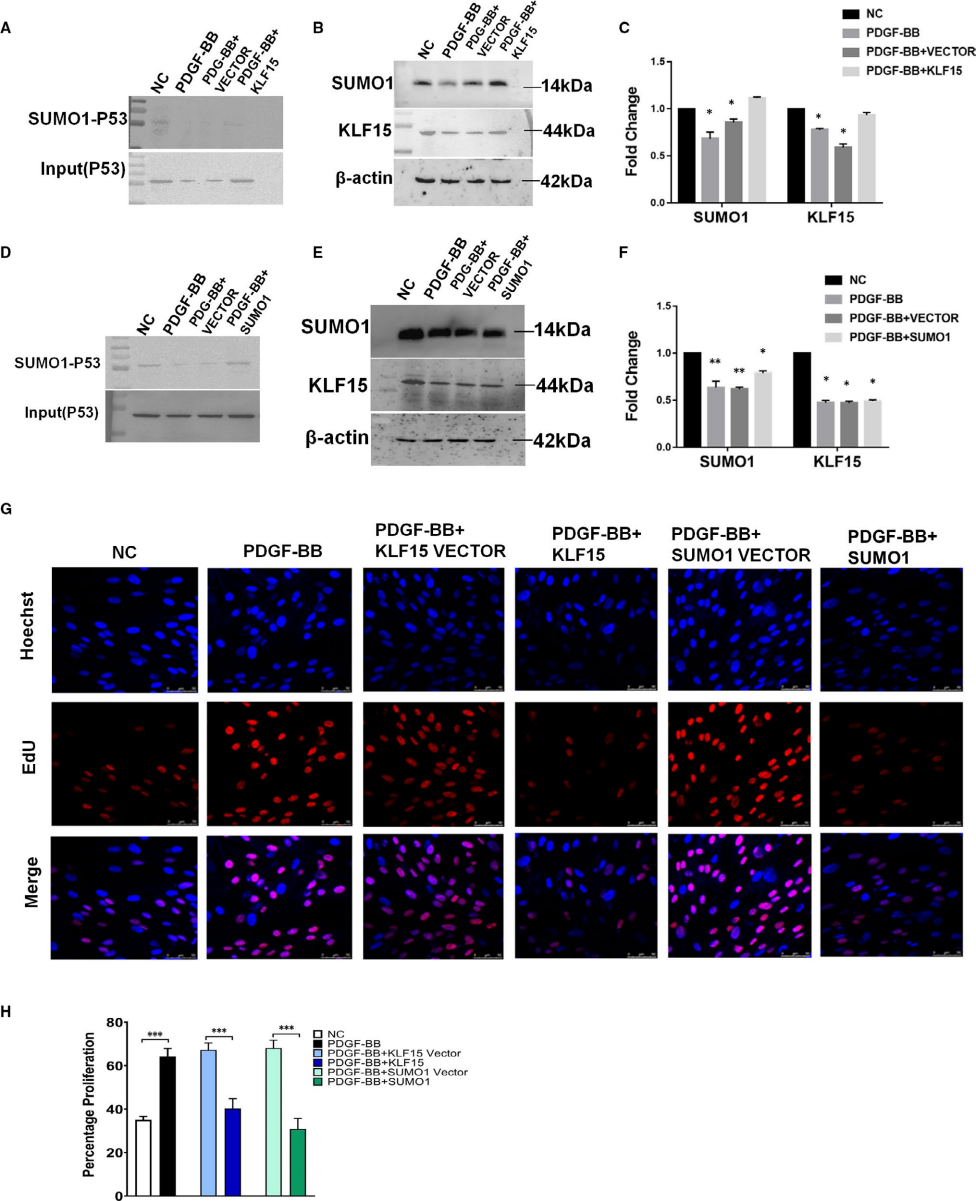
*Both KLF15 overexpression and SUMO1 overexpression inhibit PDGF‐BB‐induced cell proliferation*. A‐C, HRMCs were transfected with a control plasmid (VECTOR) or a KLF15 overexpression plasmid (KLF15) and incubated with PDGF‐BB. A, The expression of SUMO1‐P53 and input (P53) was assessed by IP analysis. B, The expression of KLF15 and SUMO1 was assessed by Western blot analysis. C, Quantitative data. D‐F, HRMCs were transfected with an empty vector (VECTOR) or a SUMO1 overexpression plasmid (SUMO1) and incubated with PDGF‐BB. D, The expression of SUMO1‐P53 and input (P53) was assessed by IP analysis. E, The expression of KLF15 and SUMO1 was assessed by Western blot analysis. F, Quantitative data. G‐H, Cell proliferation assay results. KLF15 or SUMO1 overexpression inhibited PDGF‐BB‐induced cell proliferation. *P < .05, n = 3

To establish the regulation of P53 SUMOylation and MC proliferation by KLF15 and SUMO1, we intervened with KLF15 or SUMO1 expression in HRMCs and treated HRMCs with PDGF‐BB. Among PDGF‐BB‐treated HRMCs, cells transfected with the KLF15 overexpression plasmid exhibited higher expression of SUMO1‐P53, P53, KLF15 and SUMO1 than cells transfected with the KLF15 control plasmid. The same changes in SUMO1‐P53, P53 and SUMO1 were found in the SUMO1 overexpression plasmid‐transfected cells, while KLF15 expression was unchanged in these cells (Figure 5A‐F). PDGF‐BB is one of the most effective growth factors of MCs described thus far, and a proliferative response of HRMCs to PDGF‐BB was observed. As shown in Figure 5G,H, the percentage of EdU‐positive cells was significantly increased by administration of recombinant PDGF‐BB. The EdU staining results indicated that both KLF15 and SUMO1 overexpression inhibited PDGF‐BB‐induced cell proliferation (Figure 5G,H).

**FIGURE 6:**
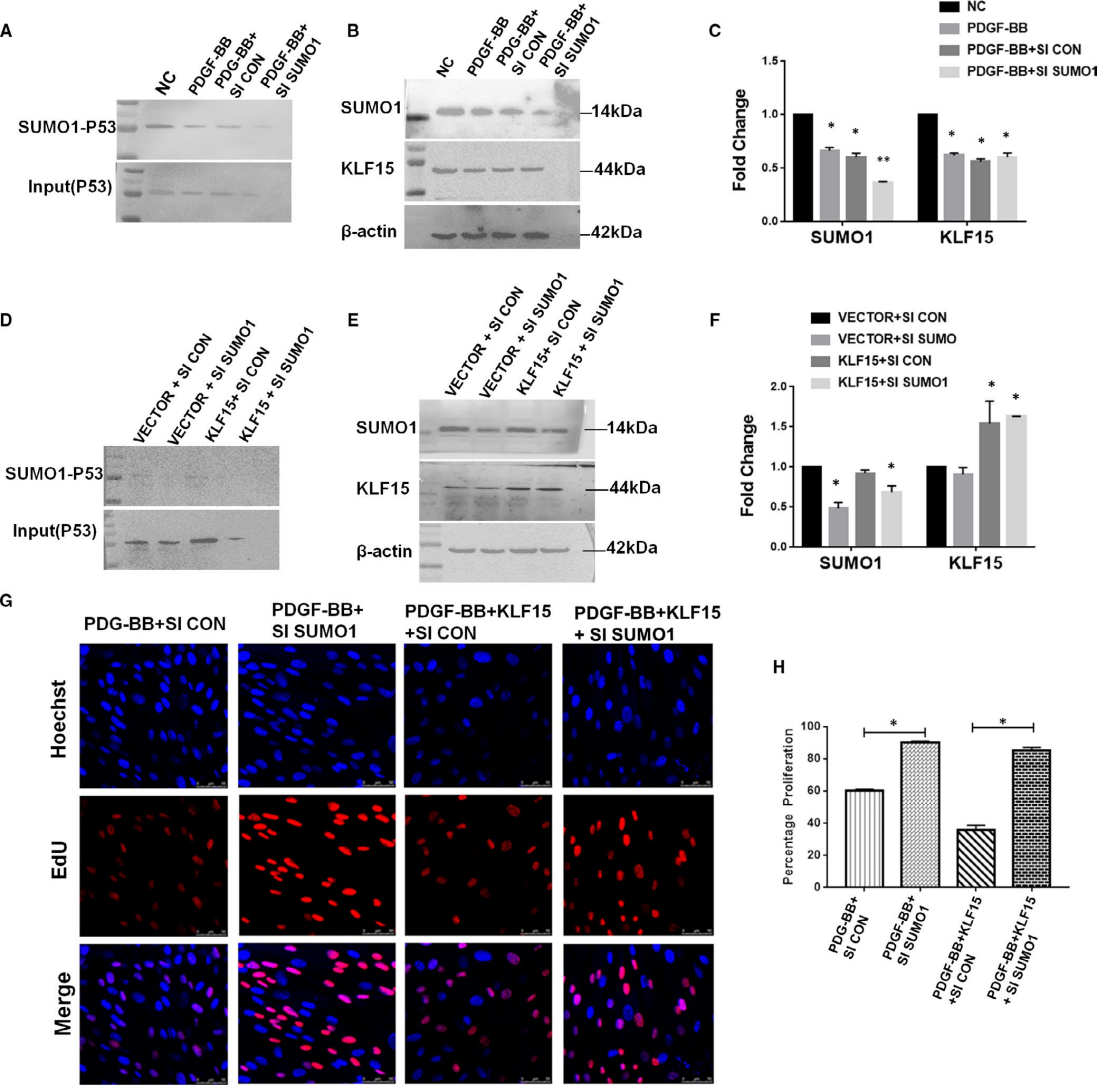
*KLF15 suppresses MC proliferation by* *enhancing p53 SUMO1 conjugation*. A‐C, HRMCs were transfected with negative control siRNA (SI CON) or SUMO1 siRNA (SI SUMO1) and incubated with PDGF‐BB. A, The expression of SUMO1‐P53 and input (P53) was assessed by IP analysis. B, The expression of KLF15 and SUMO1 was assessed by Western blot analysis. C, Quantitative data. D‐F, HRMCs were transfected with a control plasmid and negative control siRNA (VECTOR + SI CON) or SUMO1 siRNA (VECTOR + SI SUMO1), and KLF15 overexpression plasmid and negative control siRNA (KLF15 + SI CON) or SUMO1 siRNA (KLF15 + SI SUMO1) and incubated with PDGF‐BB. D, The expression of SUMO1‐P53 and input (P53) was assessed by IP analysis. E, The expression of KLF15 and SUMO1 was assessed by Western blot analysis. F, Quantitative data. G‐H, Cell proliferation assay results. *P < .05, n = 3

To gain further insight into the roles of KLF15 and SUMO1 in proliferating HRMCs, we transfected cells with only SUMO1 siRNA or cotransfected them with SUMO1 siRNA and a KLF15 overexpression plasmid. Down‐regulation of SUMO1 did not affect the expression of KLF15, but the expression levels of SUMO1‐P53 and P53 were obviously decreased after SUMO1 siRNA transfection (Figure 6A‐C). In addition, when the expression of SUMO1 was inhibited, SUMO1‐P53 and P53 expression levels were also suppressed even in cells overexpressing KLF15 (Figure 6D‐F). Then, MC proliferation was evaluated using an EdU staining assay. SUMO1 RNAi enhanced the proliferative effect of PDGF‐BB on HRMCs, and the group cotransfected with the KLF15 overexpression plasmid and SUMO1 siRNA had more EdU‐positive cells than the group cotransfected with the overexpression plasmid and the hybrid sequence control siRNA (Figure 6G,H). We therefore conclude that KLF15 suppresses MC proliferation by enhancing P53 SUMOylation.

### 3.7 Global SUMO1 and P53 expression in glomerular MCs is negatively correlated with MC proliferation in rat Thy‐1 nephritis

**FIGURE 7:**
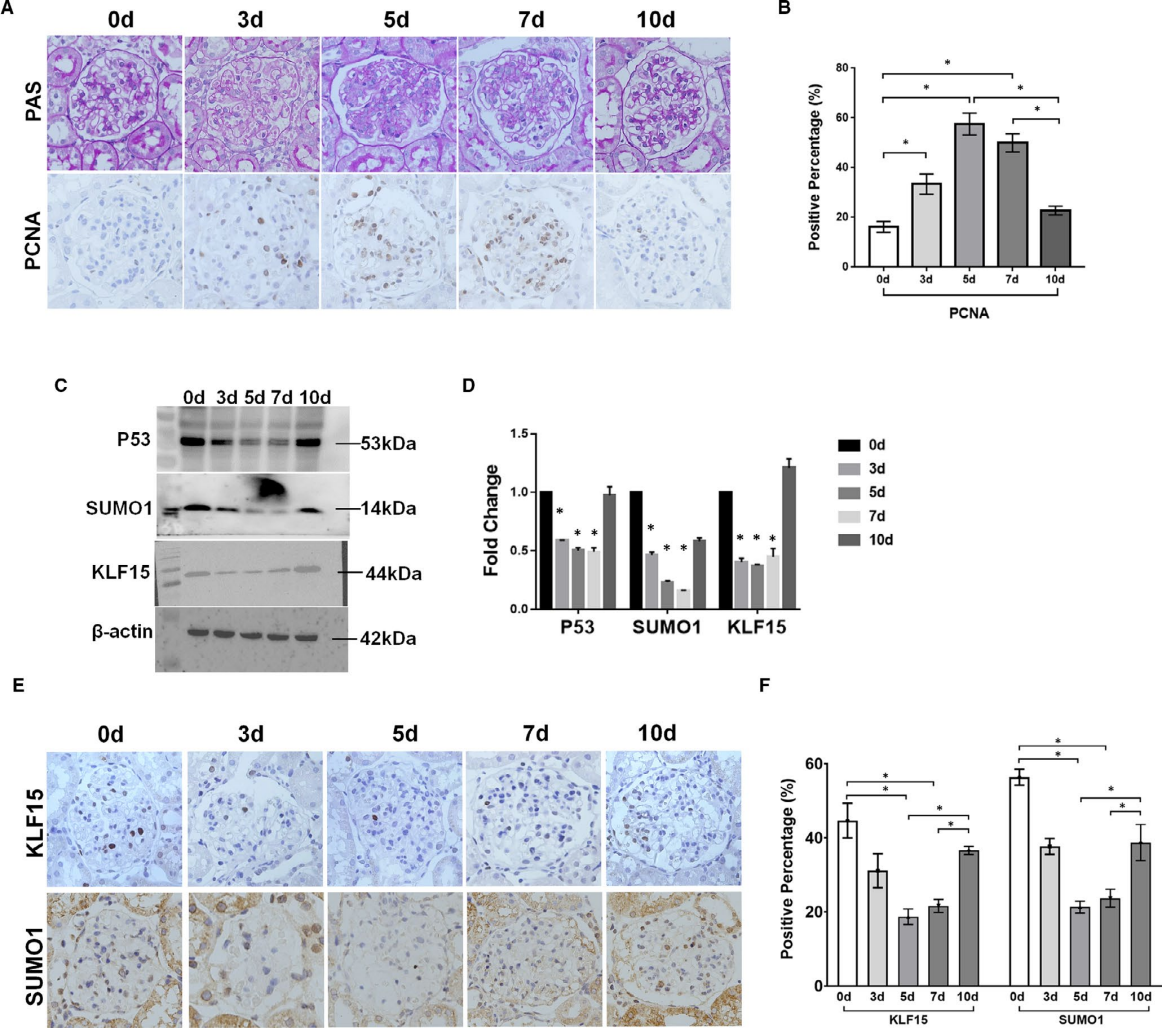
*Expression of SUMO1 and P53 in anti‐Thy1 rat glomeruli*. A, Periodic acid‐Schiff (PAS) staining results and PCNA immunohistochemical staining results. B, Statistic results of the proportion of PCNA‐positive cells in the glomerulus. C‐D, P53, KLF15 and SUMO1 expression in glomerular protein extracts. E‐F, Expression of KLF15 and SUMO1 in the glomerulus as assessed by immunohistochemical staining. *P < .05, ▲*P* < .01, #*P* < .01, n = 5

Anti‐Thy1 nephritis is a classical model of self‐limited mesangial proliferative glomerulonephritis with a proliferative phase and a recovery phase. We injected a Thy1 antibody into Wistar rats to create this model. Both serum urea nitrogen and creatinine have no significant change between control rats and the model rats (Figure S1). Marked mesangial proliferation and ECM accumulation were observed during the proliferative phase (days 5 and 7) in the model rats, and the number of MCs decreased during the recovery phase on day 10 (Figure 7A). We also detected the expression changes in the cell proliferation marker PCNA by IHC (Figure 7A,B). PCNA levels were increased on day 5, peaked on day 7 and were decreased on day 10 (Figure 7F). Western blot analysis showed that the protein expression of P53, SUMO1 and KLF15 in isolated glomeruli was lower in the model groups than in the control group (Figure 7C,D), consistent with the immunohistochemical results (Figure 7E,F). These results indicate that the abnormal proliferation of MCs in anti‐Thy1 model rats is related to the low‐level expression of KLF15 and SUMO1. Interfering with the expression of these molecules is expected to alleviate the pathological phenotype in rats.

## 4 DISCUSSION

The glomeruli are the filtration units of the kidney. Disruption of glomerular function can be caused by primary glomerular pathology or can be secondary to systemic diseases. Mesangial changes seen following glomerular injury include hyperproliferation of MCs followed by excessive production of ECM (mesangial expansion) and production of chemoattractant for inflammatory cells. Therefore, modulation of MC responses, especially abnormal proliferation, is a novel therapeutic approach. KLF15 is a protein that plays a regulatory role as a transcription factor by binding to specific DNA sequences. Many studies have reported that KLF15 is involved in the regulation of cell proliferation. For example, it has been found that KLF15 participates in the inhibition of MC proliferation‐related signalling pathways,^15^, ^30^ but its direct target gene has not been reported in the literature. Therefore, this study involved mainly joint screening of transcription and translation levels to identify the direct target gene of KLF15.

KLF15 regulates different genes in different species and in different tissues and organs. In the process of regulating the proliferation of MCs, KLF15 affects the expression of multiple genes and participates in multiple pathways.^11^, ^31^, ^32^ To explore the direct target genes of KLF15, we used ChIP‐Seq. Klein RH and his colleagues used ChIP‐seq technology to study the regulation of KLF7 on the differentiation of corneal epithelial cells.^33^ Ying M et al used this technique to study the inhibitory effect of KLF9 on the pluripotency of glioblastoma.^34^ Compared with ChIP‐chip, ChIP‐Seq enables true whole‐genome analysis with higher resolution, higher detection sensitivity and lower sample size demand.^35^ We used ChIP to obtain the DNA fragments directly bound by KLF15, and after comparison and analysis with GenBank, we screened 2478 possible target genes. Through GO and pathway analyses, we identified many target genes involved in cell cycle and proliferation processes.

ChIP‐Seq experiments require PCR for amplification of the detection signal, and some degree of bias during the amplification process is inevitable. In addition, ChIP‐Seq obtains only the genes that KLF15 can bind and does not indicate the changes that occur in the expression of these genes when the expression of KLF15 changes. We used SILAC‐LC/MS proteomics analysis to compensate for these shortcomings. This technique provides information on all the proteins whose expression changes upon regulation by KLF15, including the proteins directly regulated by KLF15 and the proteins that are indirectly regulated or post‐translationally modified. HRMCs were cultured using SILAC, and KLF15 was overexpressed in the cells through plasmid transfection. The proteins were collected for LC/MS detection, and 1357 differentially expressed proteins were obtained. GO and pathway analyses revealed that some of the differentially expressed proteins were involved in ubiquitination. The gene and protein data were intersected. The genes not related to the research purpose and genes not directly regulated by KLF15 were removed; ultimately, 52 genes were screened. Among these, the five genes with the most obvious expression differences that were the most closely related to proliferation were selected. Given the combined results of pathway analysis, SUMO1 and KLF15 expression analysis in proliferating HRMCs, ChIP‐PCR and dual‐luciferase reporter assays, the protein SUMO1 was selected as the target gene of KLF15.

In addition to ubiquitin, increasing numbers of UBL proteins,^36^, ^37^ including SUMO,^38^ neural precursor cell‐expressed, developmentally down‐regulated 8 (NEDD8) ^39^, ^40^ and interferon‐stimulated gene 15 (ISG15),^41^ are being identified. These modifying proteins powerfully regulate a variety of biological processes. The covalent addition of SUMO to substrates, termed SUMOylation, is a post‐translational modification involved in a series of cellular processes, including nuclear‐cytosolic transport, transcriptional regulation, apoptosis, protein stability control, stress responses and cell cycle progression.^27^ SUMO is typically attached to acceptor lysine residues of protein substrates harbouring a consensus sequence and contributes to the regulation of protein‐protein interactions as well as to subcellular compartmentalization and protein stability.^42^ SUMO1, as a member of SUMOS family, can affect cell proliferation by modifying substrate and stabilizing cell cycle regulatory proteins.^43^ Therefore, combined with the literature report and the comprehensive screening and analysis results, we focused on how KLF15 regulates the effect of SUMO1 on mesangial cell proliferation.

To identify the substrates of SUMO1, we performed network analysis of SUMO1 using the IPA database and SUMOsp software. Combined with published research on P53, our initial screening data suggested P53 as a downstream molecule of SUMO1 with the SUMOylation sequence Ψ‐K‐x‐D/E. Previous studies have shown that P53 was a substrate of SUMOylation,^44^, ^45^ and enhanced P53 SUMO1 conjugation promoted the stability and activity of P53 and induced senescence.^46^ In our study, interference with the expression of SUMO1 in HRMCs produced the same change trends in SUMO1‐P53 and P53, indicating that P53 was a SUMOylation substrate of SUMO1.

Emerging evidence has indicated that SUMOylation and deSUMOylation play roles in numerous nephropathic diseases, such as renal dysgenesis, renal carcinoma, glomerular disease, podocyte apoptosis, renal medulla hypertonicity, acute kidney injury and nephrolithiasis.^47^, ^48^, ^49^, ^50^, ^51^ To clarify the relationship among KLF15, SUMO1, P53 SUMOylation and MC proliferation, we performed a series of transfection treatments on cells that had undergone PDGF‐BB‐induced proliferation. Overexpression of KLF15 up‐regulated the expression of SUMO1, while overexpression of SUMO1 did not affect the expression of KLF15. Either KLF15 or SUMO1 overexpression increased the SUMOylation of P53 and antagonized the cell proliferation induced by PDGF‐BB. When the expression of SUMO1 was inhibited, the SUMOylation of P53 was also inhibited, and KLF15 lost its antagonistic effect on cell proliferation. In vivo, the proliferation of MCs gradually increased with aggravation of mesangial proliferative nephritis, while the expression of KLF15, SUMO1 and P53 decreased significantly. Considering the integrated observations in cells and tissues, we preliminarily conclude that KLF15 regulates the SUMOylation of P53 through SUMO1, thereby inhibiting MC proliferation.

## 5 CONCLUSIONS

Some studies have indicated that KLF15 plays an important role in regulating the proliferation of MCs. In this work, we explored the mechanisms of MC proliferation suppression mediated by this transcription factor. We identified SUMO1 as the primary target of KLF15 in MCs via bioinformatics analyses involving ChIP‐Seq and SILAC‐LC/MS. Furthermore, with the aid of the IPA database and SUMOsp software, we determined that P53 was a direct substrate of SUMO1. In vitro and in vivo experiments confirmed that KLF15 could promote the expression of SUMO1 and the SUMOylation of P53 then inhibit the proliferation of MCs (Figure S2). These results contribute to the understanding of the regulatory role of KLF15 in MC proliferation and provide a theoretical basis for finding new treatments for MC proliferation‐related kidney diseases.

## CONFLICT OF INTEREST

The authors confirm that there are no conflicts of interest.

## AUTHOR CONTRIBUTIONS

**Lingling Wu:** Formal analysis (lead); Methodology (equal); Writing‐original draft (lead). **Ou Li:** Data curation (equal); Methodology (equal); Writing‐original draft (equal). **Fengge Zhu:** Validation (equal). **Pu Chen:** Methodology (supporting). **Xu Wang:** Formal analysis (equal); Methodology (supporting). **Guangyan Cai:** Supervision (supporting). **Xiangmei Chen:** Conceptualization (supporting); Writing‐review & editing (supporting). **Quan Hong:** Conceptualization (lead); Project administration (lead); Supervision (lead); Writing‐original draft (lead); Writing‐review & editing (lead).

## Supporting information

Figure S1‐2
