# Supplementary material for: Krϋppel‐like factor 15 suppresses renal glomerular mesangial cell proliferation via enhancing P53 SUMO1 conjugation
Source: J Cell Mol Med. 2021 May 4;25(12):5691–706. doi: 10.1111/jcmm.16583 (PMC8184688; doi:10.1111/jcmm.16583)
Supplement: Supplementary file 1 — Figure S1‐2 [file JCMM-25-5691-s001.docx]

**Supplementary materials**

**
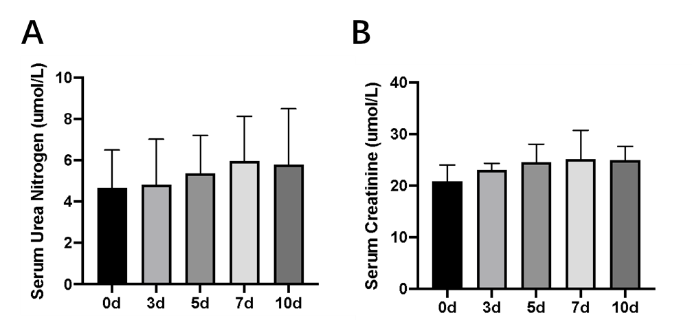
**

**Figure S1.** *Serum urea nitrogen and creatinine test results.* A: Serum urea nitrogen result. B: Creatinine result. n=5.

**
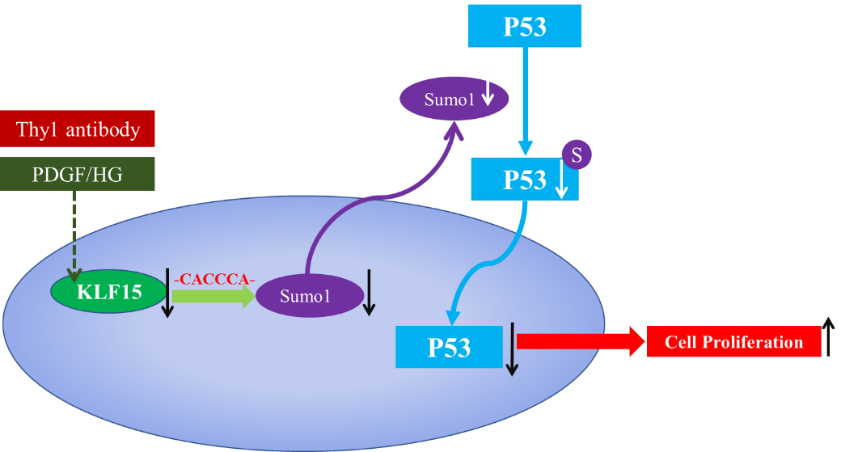
**

**Figure S2.** *Graphic overview of the mechanism by which KLF15 regulates the proliferation of MCs.* In the high glucose (HG) or PDGF stimulation environment, decreased KLF15 expression of mesangial cell would promote the SUMO1 transcript via binding the consensus sequence of the promoter. Downregulated SUMO1 decreased the sumoylation of p53 then exacerbate the cell proliferation.
